# Balancing accuracy, completeness, and efficiency for rice 3D reconstruction through CBAM-UNet-based multi-view segmentation and camera configuration optimization

**DOI:** 10.3389/fpls.2026.1882534

**Published:** 2026-09-24

**Authors:** Haoyang Zhou, Rongjie Chen, Hao Wang, Minglu Li, Yongkang Teng, Shenghao Ye, Menglei Wei, Kun Yu, Pingping Fang, Jing Cao, Fanglin Zhu, Wenyu Zhang, Ting Sun, Min Jiang

**Affiliations:** 1 Cross-Strait Agricultural Technology Cooperation Center of Ministry of Agriculture and Rural Affairs, Key Laboratory of Ministry of Education for Genetics, Breeding and Multiple Utilization of Crops, College of Agriculture, Fujian Agriculture and Forestry University, Fuzhou, China; 2 Jiangsu Academy of Agricultural Sciences Wuxi Branch, Wuxi, China; 3 School of Agricultural Engineering, Jiangsu University, Zhenjiang, China; 4 Institute of Germplasm Resources and Biotechnology, Jiangsu Academy of Agricultural Sciences, Nanjing, China; 5 Quzhou Key Laboratory for Germplasm Innovation and Utilization of Crop, Quzhou Academy of Agricultural and Forestry Sciences, Quzhou, China; 6 Institute of Agricultural Economics and Development, Jiangsu Academy of Agricultural Sciences, Nanjing, China

**Keywords:** 3D reconstruction, camera configuration, crop phenotyping, image segmentation, rice

## Abstract

Multi-view 3D reconstruction has been widely applied in plant phenotyping, but the complex canopy structure of rice plants poses significant challenges for reconstruction accuracy, completene7ss, and efficiency. In this study, we developed an optimized workflow for 3D reconstruction of rice using the self-developed Metatlas V1 multi-view imaging platform combined with the COLMAP + OpenMVS pipeline. A CBAM-UNet-based image segmentation model was used to extract plant regions from complex backgrounds, outperforming thresholding, U-Net, and U-Net++, and increasing the number of reconstructed points. Camera configuration optimization was performed by systematically combining equidistant and greedy strategies, resulting in an optimal six-camera setup (#2, #3, #4, #6, #7, and #8, corresponding to −30°, −15°, 0°, +30°, +45°, and +60° relative to the horizontal viewpoint), which provided complementary views of the canopy inner structure and stem base. This configuration reduced the average nearest-neighbor distance to 0.037–0.066 cm across different varieties and growth stages compared with the full 11-camera setup, retained 73–76% of the points, and decreased reconstruction duration by approximately 70–80%. The strong agreement between point-cloud-derived and manually measured plant height and canopy width (*R*^2^ = 0.989 and 0.946, respectively) supported the accuracy of the point-cloud-derived phenotypic measurements. Overall, integrating image segmentation with camera-configuration optimization offers an accurate and efficient solution for high-throughput 3D phenotyping of rice using Metatlas V1, balancing reconstruction accuracy, point-cloud completeness, and computational efficiency, and may provide a methodological reference for camera-configuration design in other rotational multi-view phenotyping platforms.

## 1 Introduction

Recent advances in agricultural science and information technologies have propelled plant phenotyping into the high-throughput era (Song et al., 2021; Liu et al., 2025). High-throughput phenotyping (HTP) enables rapid, non-destructive acquisition of extensive data on plant morphological, physiological, and biochemical traits, thereby establishing a robust framework for crop growth monitoring, germplasm screening, and genotype-phenotype association studies (Jangra et al., 2021; Persa et al., 2021; Gano et al., 2024; Sun et al., 2024). A wide range of optical sensing approaches, including RGB, multispectral, near-infrared (NIR), fluorescence, and hyperspectral imaging, have been extensively utilized for HTP (Sun et al., 2022; Zhang et al., 2023b). Moreover, emerging wearable sensor technologies have facilitated *in situ* and continuous phenotyping (Zhang et al., 2023a; Sun et al., 2025). Nevertheless, the phenotypic information derived from these approaches remains predominantly two-dimensional (2D) at the spatial scale of the target crop, limiting their capacity to accurately capture canopy architecture such as leaf inclination and azimuth angle (Harandi et al., 2023; Rui et al., 2024).

In recent years, both terrestrial and airborne LiDAR technologies have been widely employed for three-dimensional (3D) crop reconstruction; however, their relatively high equipment cost, operational complexity, and the sensitivity of point-cloud quality to scanning geometry and canopy occlusion may constrain their broader adoption in agriculture (Guo et al., 2018; Hu et al., 2022; Gu et al., 2024). With the continual reduction in industrial camera costs and the rapid advancement of computational capabilities, multi-view imaging has emerged as an accurate and cost-effective alternative for crop 3D reconstruction (Akhtar et al., 2024; Yang et al., 2024). Multi-view reconstruction techniques can be broadly classified into explicit modeling, which represents scenes through defined geometric data structures such as point clouds, meshes, or voxels exemplified by the Structure from Motion and Multi-View Stereo (SfM-MVS) pipeline (El Hazzat and Merras, 2023), and implicit modeling, which encodes geometry using mathematical functions or neural representations as in Neural Radiance Fields (NeRF) (Akhtar et al., 2024). The SfM-MVS pipeline generally includes feature detection, keypoint correspondence, identifying geometrically consistent matches, structure from motion, scale and georeferencing, refinement of parameter values, and multi-view stereo image matching algorithms (Wang et al., 2020). Compared with implicit methods, the SfM-MVS pipeline offers higher interpretability and benefits from a well-established software ecosystem, rendering it the predominant approach for plant 3D phenotyping (Mach et al., 2025).

Despite its widespread adoption, achieving high-precision and computationally efficient SfM-MVS reconstruction remains challenging. Image preprocessing, feature detection and matching, depth-map fusion, and pipeline integration are employed to enhance the reconstruction accuracy and computational efficiency of the SfM-MVS pipeline (Ioannidis et al., 2025). Among these, image preprocessing is a critical step for ensuring input image quality and robust feature matching, encompassing both image segmentation and camera configuration. Previous studies have shown that denoising, background subtraction, and region-of-interest (ROI) extraction can significantly improve dense point-cloud yield and reconstruction quality (Zhu et al., 2020; He et al., 2023). The rice canopy, characterized by extensive tillering and long, slender, overlapping leaves, presents greater challenges for 3D reconstruction. Compared to crops such as maize (Wu et al., 2020) and soybean (Sun et al., 2023), rice 3D reconstruction requires more viewpoints and higher-quality images, which inevitably increases computational demands and reconstruction time. Therefore, it is crucial to balance reconstruction accuracy and efficiency through image segmentation and optimization of camera poses and numbers.

To address these challenges, we acquired high-quality multi-view images covering a wide range of viewpoints for rice plants at different growth stages using the self-developed Metatlas V1 platform and conducted an optimization study of the image preprocessing workflow. A CBAM-UNet segmentation network was developed by embedding channel and spatial attention modules (CBAM) into the U-Net skip connections to enhance salient feature extraction and fine-detail representation, thereby improving segmentation accuracy and robustness under complex backgrounds. Camera configurations, including variations in poses and numbers, were compared and optimized using Equidistant and Greedy Algorithm strategies. Our study provides an effective approach for balancing reconstruction accuracy and efficiency in SfM-MVS pipeline-based rice 3D reconstruction.

## 2 Materials and methods

### 2.1 Experiment design

The experiment was carried out from May to October 2024 at Jiangsu Academy of Agricultural Sciences Wuxi Branch (120.29°E, 31.63°N). Four rice cultivars with distinct plant architectures were used as experimental materials: Hejiang18, Zhongzu143, Nanjing9108, and Guangluai4. Rice seeds were sown on May 15 and grown under dry nursery conditions until the three-leaf stage. On June 18, the seedlings were transplanted into plastic pots (30 cm in height and 33 cm in outer diameter), each containing 15 kg of dry clay loam. Each pot contained one hill consisting of a single plant. Nine plants were grown for each cultivar, resulting in 36 plants in total. At the heading stage, six plants per cultivar exhibiting normal and representative growth and no visible pest or disease damage were selected and labeled R1–R6. These 24 plants, each considered an independent biological replicate, were imaged at both the heading and maturity stages. At the end of tillering, ineffective tillers were suppressed by midseason drainage, and thereafter a 2–5 cm water depth was maintained until one week before maturity, when irrigation was stopped. Prior to transplanting, 8 g of compound fertilizer (15-15-15) was applied per pot as basal fertilizer. Subsequently, 1.9 g of urea was applied as tillering fertilizer, and at the jointing stage, 1.9 g of urea and 1 g of KCl were applied as panicle fertilizer, corresponding to field application rates of 20 kg N, 8 kg P_2_O_5_, and 12 kg K_2_O per mu. Weed, pest, and disease management followed local rice cultivation practices to minimize biotic and abiotic stresses.

### 2.2 Multi-view imaging

To acquire multi-view images of rice plants, we employed the self-developed crop 3D phenotyping platform Metatlas V1 (**Figure 1**). The platform consists of a base, a rotary unit (including gears, a turntable, and an imaging arm), camera modules, an industrial computer (IPC), a programmable logic controller (PLC), a servo motor, and a 48 V DC power supply (**Figure 1a**). The imaging arm comprises a 2.1-m-high perforated vertical column and adjustable horizontal telescopic rods with lengths ranging from 0.5 to 1.3 m. In the configuration used in this study, the arm accommodated 11 cameras mounted at different heights along the column. The correspondence between the camera position indices shown in **Figure 1a** and their viewing angles is provided in **Supplementary Table S1**. Camera positions were indexed from 0 to 10, representing a total of 11 camera positions. Each camera module features a 50-megapixel sensor with autofocus, auto-exposure, and rolling shutter, and an imaging distance ranging from 10 cm to infinity.

**Figure 1:**
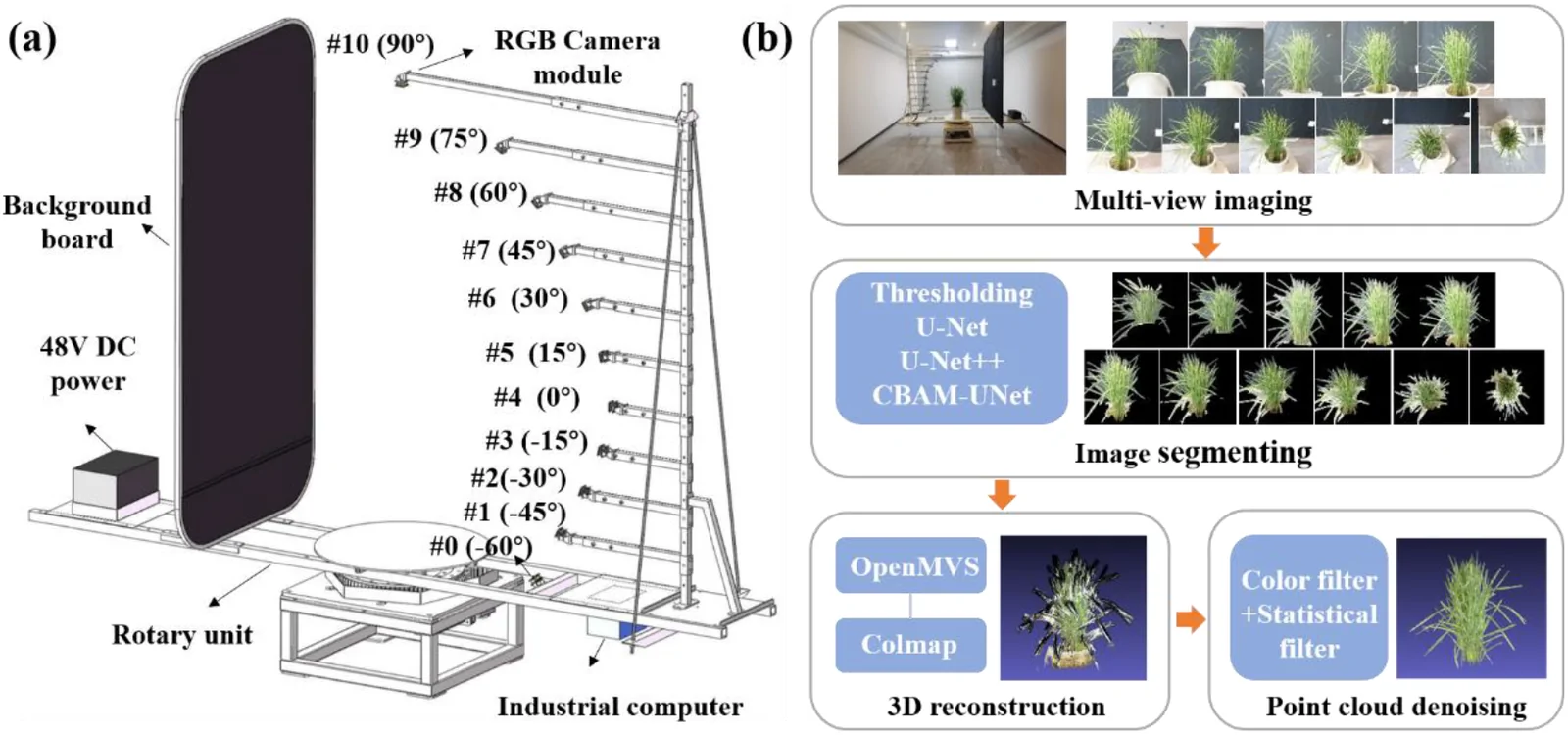
3D reconstruction of rice plants based on multi-view images. **(a)** Self-developed crop 3D phenotyping platform, Metatlas V1. The cameras were sequentially numbered from #0 to #10 from bottom to top, with camera #4 representing the horizontal viewpoint. The angular separation between adjacent cameras was set to 15°. **(b)** Workflow of 3D reconstruction for rice plants, encompassing multi-view image acquisition using the Metatlas V1 platform, image segmentation with thresholding, U-Net, U-Net++, and CBAM-UNet models to obtain binary masks, generation of masked RGB images retaining plant texture against a black background, 3D reconstruction through the COLMAP + OpenMVS pipeline, and subsequent point cloud denoising employing color filtering and statistical filtering.

The rotation parameters of the platform (e.g., motor speed, direction, and angular displacement per image) are controlled in real time by the self-developed software via PLC communication. At each predefined azimuthal position, the system automatically triggers the cameras to capture multi-view image sequences, after which the platform rotates to the next position. In this study, all cameras captured images at horizontal intervals of 15°, with the platform stopping at each position to acquire multi-view image sequences, resulting in a total of 24 captures. The control system coordinates turntable rotation, image acquisition, data storage, and transmission to enable automated multi-view image acquisition.

### 2.3 Multi-view image segmentation

#### 2.3.1 Dataset preparation

Multi-view images of rice plants were manually annotated using the LabelMe software (MIT CSAIL, USA) (**Supplementary Figure S1**). Polygonal masks were drawn around each rice plant to precisely delineate plant regions from the background (**Supplementary Figure S1a**). The annotated images were converted into binary masks, where the pixel values of rice plant regions were set to 1, and those of the background were set to 0 (**Supplementary Figure S1b**). The resulting dataset comprised 600 annotated images from 24 plants, with six plants per cultivar imaged at both growth stages. For each cultivar–stage combination, the training set contained 60 images from plants R1–R5. Specifically, 55 images were obtained by imaging each plant at one randomly selected horizontal viewing position using all 11 vertical camera positions (#0–#10), and five additional images of R1 were obtained at a second horizontal viewing position using camera positions #1, #3, #4, #6, and #9. This resulted in 480 training images.

Plant R6 of each cultivar was used as the validation set. For each cultivar–stage combination, 11 images of R6 were acquired at one horizontal viewing position using all camera positions (#0–#10), and four additional images were acquired at a second horizontal viewing position using positions #1, #4, #6, and #9, yielding 15 validation images per combination and 120 validation images in total. The dataset was therefore partitioned into training and validation sets at the plant level, yielding an 80:20 training-to-validation ratio by image count. All images from R1–R5 were assigned to the training set, whereas all images from R6 were assigned to the validation set, ensuring plant-level separation between the two subsets. The same fixed partition was used for all segmentation models, and the detailed dataset composition is provided in **Supplementary Table S2**.

#### 2.3.2 Segmentation model

To address the challenges of variable plant morphology, complex backgrounds, and illumination differences in rice canopy imagery, we adopted a CBAM-UNet segmentation model (**Figure 2**). The architecture is based on the classical U-Net network (Ronneberger et al., 2015), which consists of an encoder–decoder structure and skip connections, and is widely used in semantic segmentation tasks. The standard U-Net was enhanced by integrating the Convolutional Block Attention Module (CBAM) Woo et al., 2018) into each skip connection (**Figure 2a**). The CBAM module consists of a Channel Attention Module (CAM) and a Spatial Attention Module (SAM), which sequentially refine feature maps by emphasizing informative channels and critical spatial regions, thereby improving segmentation accuracy under varying illumination and background conditions (**Figure 2b**).

**Figure 2:**
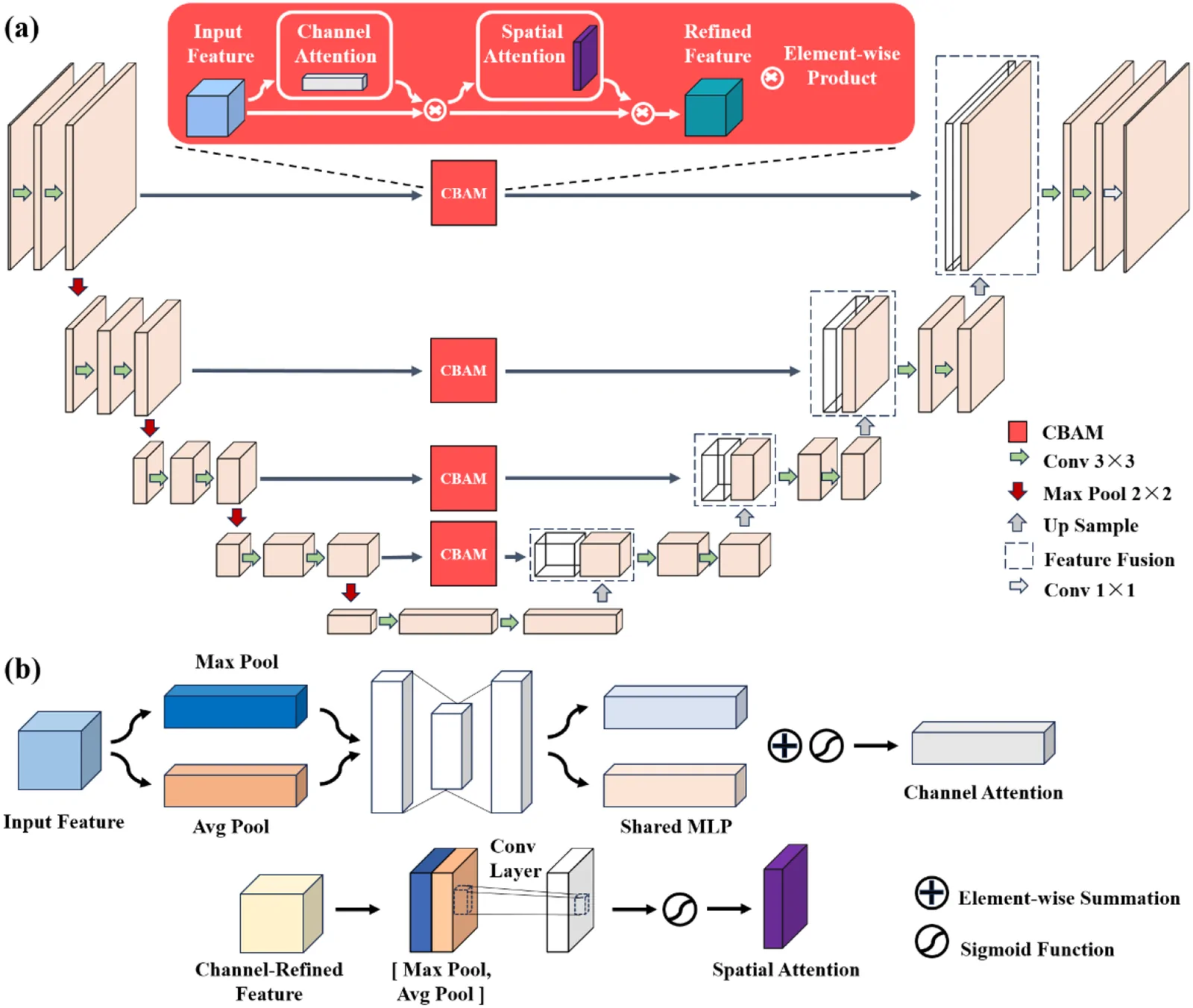
Structural diagram of the CBAM-UNet model. **(a)** Overall architecture of the CBAM-UNet. The model follows the encoder–decoder structure of U-Net, where the encoder progressively extracts multi-scale features through convolution and pooling operations, while the decoder restores spatial resolution via upsampling and feature fusion. Convolutional Block Attention Modules (CBAM) are integrated into each skip connection to adaptively refine and highlight informative features transmitted from the encoder to the decoder. **(b)** Structure of the Convolutional Block Attention Module (CBAM). The CBAM sequentially applies a Channel Attention Module (CAM) and a Spatial Attention Module (SAM). The CAM captures inter-channel dependencies by applying global max-pooling and average-pooling operations followed by a shared multilayer perceptron (MLP) to produce channel-wise attention weights. The SAM generates spatial attention maps by convolving concatenated pooled features, emphasizing critical spatial regions in the feature map. The refined feature is obtained by element-wise multiplication of the input feature with both attention maps.

For comparison, three additional segmentation methods were tested: simple thresholding, U-Net, and U-Net++ (**Figure 1b**). Thresholding distinguished plant regions based on pixel color values. U-Net utilized an encoder–decoder framework with skip connections to capture plant structures across multiple scales. U-Net++ further extended U-Net through dense, multi-level skip connections between encoder and decoder pathways, enhancing feature propagation and fusion for more accurate representation of fine leaf structures and complex canopy morphology.

#### 2.3.3 Model implementation

The segmentation model was implemented in Python 3.8 using the PyTorch 1.10.0 framework with CUDA 11.3 support. All experiments were performed on a Windows 11 workstation equipped with an Intel^®^ Core™ i9-14900KF CPU (3.20 GHz), 128 GB RAM, and an NVIDIA RTX 4070 GPU (12 GB VRAM). During segmentation, the original RGB images were loaded using OpenCV, resized to 512 × 512 pixels using bilinear interpolation, and converted from BGR to single-channel grayscale images using cv2.COLOR_BGR2GRAY. The grayscale values were converted to float32 and retained in the 0–255 range without normalization or test-time augmentation. The output logits were directly thresholded at 0.5 to generate binary masks (plant = 255; background = 0), which were restored to the original resolution using nearest-neighbor interpolation. During model development, an initial CBAM-UNet run was conducted for 80 epochs to assess convergence. The model was trained using the RMSProp optimizer with an adaptive learning rate initialized at 0.00001 and a batch size of 1. To train the segmentation model, the Binary Cross-Entropy with Logits Loss was adopted, which combines the sigmoid activation and binary cross-entropy loss in a numerically stable formulation. The loss for each sample can be expressed as Equation 1:

(1) $$L(x,y)=\max(x,0)-x\cdot y+\text{log}(1+{\text{e}}^{-|x|})$$

where *x* denotes the predicted logit, and *y*∈ {0,1} represents the ground-truth label.

To assess run-to-run variability, U-Net and CBAM-UNet were each trained and evaluated across three independent runs using the same data partition and protocol: one run without a prespecified random seed and two runs with seeds of 42 and 123. A seed-42 ablation compared U-Net, U-Net + CAM, U-Net + SAM, and CBAM-UNet, with CAM, SAM, or CBAM inserted into the four skip connections before the decoder. Model complexity and inference efficiency were evaluated using PyTorch 2.5.1 with CUDA 12.1 on the same workstation. FLOPs were calculated for a single forward pass with a 512 × 512 input and a batch size of 1, peak GPU memory was measured using torch.cuda.max_memory_allocated(), and mean inference time was calculated from 100 synchronized forward passes after 10 warm-up passes, excluding image loading and preprocessing.

#### 2.3.4 Evaluation metrics

In this study, the mean intersection over union (*mIoU*), mean pixel accuracy (*mPA*), and F1-score (*F1*) were employed to evaluate the performance of the segmentation models. These evaluation metrics are computed as shown in Equations 2–6:

(2) $$mIoU=\frac{1}{C}\sum_{i=1}^{C}\frac{TP_{\text{i}}}{TP_{\text{i}}+FP_{\text{i}}+FN_{\text{i}}}$$

(3) $$mPA=\frac{1}{C}\sum_{i=1}^{C}\frac{TP_{\text{i}}}{TP_{\text{i}}+FN_{\text{i}}}$$

(4) $$F1=\frac{2\times Pr\text{e}c\times Rec}{Pr\text{e}c+Rec}$$

(5) $$Pr\text{ec}=\frac{TP}{TP+FP}$$

(6) $$Rec=\frac{TP}{TP+FN}$$

where *C* is the total number of classes, *TP*_i_​, *FP*_i_, and *FN*_i_​ denote the numbers of true positive, false positive, and false negative pixels for class *i*, respectively. For binary classification, the precision (*Prec*) and recall (*Rec*) represent the ratios of correctly predicted positive pixels to all predicted and actual positive pixels, respectively. The reported *mIoU* and *mPA* were macro-averaged across the background and plant classes. Plant-class *IoU*, *Prec*, and *Rec* were additionally calculated from the aggregated pixel-level confusion matrix.

#### 2.3.5 Image post-processing

The segmentation outputs were binary masks. During inference, a copy of each original 6120 × 8160-pixel RGB image was processed as described in Section 2.3.3 to generate a 512 × 512 single-channel input without preserving the original aspect ratio. Each predicted binary mask was subsequently resized to 6120 × 8160 pixels using nearest-neighbor interpolation and applied pixelwise to the corresponding original RGB image. Pixels outside the predicted plant region were set to black, whereas the original RGB values within the plant region were retained. Thus, the outputs of this step were masked RGB images rather than binary images, and these masked RGB images were used as inputs to the SfM-MVS pipeline. Because the RGB images used for reconstruction were not resized or cropped, they retained the original dimensions, aspect ratio, and pixel coordinate system; therefore, no additional rescaling of the camera intrinsics was required.

### 2.4 3D reconstruction and denoising

Images of four rice varieties at the heading and maturity stages, which were not included in the training or validation sets, were used for 3D reconstruction of the rice plants. The reconstruction was performed using an open-source pipeline combining COLMAP (version 3.10) (Schonberger and Frahm, 2016) for sparse reconstruction and camera pose estimation, and OpenMVS (version 2.1.0; https://github.com/cdcseacave/openMVS) for dense MVS reconstruction (**Figure 1b**). All reconstruction steps were performed using default parameters. Keypoints and descriptors were first extracted from the masked RGB images, and feature correspondences were established across multiple views. COLMAP used the SIMPLE_RADIAL camera model; camera intrinsics and extrinsics were estimated and refined through bundle adjustment to generate a sparse 3D point cloud of the rice plant. The sparse reconstruction and estimated camera parameters were then fed into OpenMVS, where depth maps were computed for each view and subsequently fused to generate a dense 3D point cloud capturing the detailed structure of the plant canopy.

Dense point clouds often contained noise and small artifacts, which could interfere with downstream analyses. To address this, a two-step denoising procedure was applied (**Figure 1b**). First, point colors were converted from the RGB color space to the HSV color space using OpenCV, and points outside the predefined HSV ranges were removed. The thresholds were set to 0–179 for hue, 43–255 for saturation, and 140–255 for value, following the OpenCV HSV scale. Second, statistical outlier removal was performed using 20 nearest neighbors, and points whose mean distance to neighboring points exceeded 2.0 standard deviations from the global mean were removed. This workflow effectively produced clean and complete point clouds of individual rice plants, suitable for quantitative structural analysis and high-throughput 3D phenotyping.

### 2.5 Camera configuration optimization

Multi-view images captured by 11 cameras on the Metatlas V1 platform were used to optimize camera number and pose. Cameras were numbered #0 to #10 from bottom to top, with camera #4 corresponding to the horizontal (0°) viewpoint. Adjacent cameras were separated by 15°, giving elevation angles of -60°, -45°, -30°, -15°, 0°, +15°, +30°, +45°, +60°, +75°, and +90°for cameras #0 to #10, respectively. Candidate configurations were generated using equidistant and greedy strategies. Three evaluation dimensions—accuracy, completeness, and efficiency—were considered. Within the camera-configuration comparison, accuracy was operationally assessed as relative geometric agreement with the internal 11-camera reference using the average nearest-neighbor distance (ANND), rather than as absolute geometric accuracy. Completeness was indirectly assessed using total point count as a descriptive indicator of point-cloud yield, rather than as an independent metric directly measuring structural completeness. Computational efficiency was evaluated using reconstruction time.

#### 2.5.1 Equidistant strategy

Because feature extraction and matching deteriorate when the angular gap between adjacent cameras exceeds 60° (Wu, 2013), the maximum interval was set to 60°. Under the equidistant strategy, cameras were arranged so that the angle between each selected camera and its neighboring camera, relative to the center of the imaging area, was equal. Four interval angles of 15°, 30°, 45°, and 60° were selected to construct camera configurations. The number of cameras was gradually increased from 1 to 11, forming 11 primary groups of configurations. Within each group, camera IDs were selected according to the equidistant strategy based on the interval angle. Using this systematic approach, a total of 116 distinct camera configurations were generated, reflecting the practically achievable positions of the 11 cameras (**Table 1**).

**Table 1:** Camera configuration based on equidistance strategy.

Number of cameras	Interval angle	Camera IDs
1	15°	NO. 0; NO. 1; NO. 2; NO. 3; NO. 4; NO. 5; NO. 6; NO. 7; NO. 8; NO. 9; NO. 10
2	15°	NO. 0 & 1; NO. 1 & 2; NO. 2 & 3; NO. 3 & 4; NO. 4 & 5; NO. 5 & 6; NO. 6 & 7; NO. 7 & 8; NO. 8 & 9; NO. 9 & 10
	30°	NO. 0 & 2; NO. 1 & 3; NO. 2 & 4; NO. 3 & 5; NO. 4 & 6; NO. 5 & 7; NO. 6 & 8; NO. 7 & 9; NO. 8 & 10
	45°	NO. 0 & 3; NO. 1 & 4; NO. 2 & 5; NO. 3 & 6; NO. 4 & 7; NO. 5 & 8; NO. 6 & 9; NO. 7 & 10
	60°	NO. 0 & 4; NO. 1 & 5; NO. 2 & 6; NO. 3 & 7; NO. 4 & 8; NO. 5 & 9; NO. 6 & 10;
3	15°	NO. 0 & 1 & 2; NO. 1 & 2 & 3; NO. 2 & 3 & 4; NO. 3 & 4 & 5; NO. 4 & 5 & 6; NO. 5 & 6 & 7; NO. 6 & 7 & 8; NO. 7 & 8 & 9; NO. 8 & 9 & 10
	30°	NO. 0 & 2 & 4; NO. 1 & 3 & 5; NO. 2 & 4 & 6; NO. 3 & 5 & 7; NO. 4 & 6 & 8; NO. 5 & 7 & 9; NO. 6 & 8 & 10
	45°	NO. 0 & 3 & 6; NO. 1 & 4 & 7; NO. 2 & 5 & 8; NO. 3 & 6 & 9; NO. 4 & 7 & 10;
	60°	NO. 0 & 4 & 8; NO. 1 & 5 & 9; NO. 2 & 6 & 10
4	15°	NO. 0-3; NO. 1-4; NO. 2-5; NO. 3-6; NO. 4-7; NO. 5-8; NO. 6-9; NO. 7-10
	30°	NO. 0 & 2 & 4 & 6; NO. 1 & 3 & 5 & 7; NO. 2 & 4 & 6 & 8; NO. 3 & 5 & 7 & 9; NO. 4 & 6 & 8 & 10;
	45°	NO. 0 & 3 & 6 & 9; NO. 1 & 4 & 7 & 10;
5	15°	NO. 0-4; NO. 1-5; NO. 2-6; NO. 3-7; NO. 4-8; NO. 5-9; NO. 6-10
	30°	NO. 0 & 2 & 4 & 6 & 8; NO. 1 & 3 & 5 & 7 & 9; NO. 2 & 4 & 6 & 8 & 10;
6	15°	NO. 0-5; NO. 1-6; NO. 2-7; NO. 3-8; NO. 4-9; NO. 5-10
	30°	NO. 0 & 2 & 4 & 6 & 8 & 10
7	15°	NO. 0-6; NO. 1-7; NO. 2-8; NO. 3-9; NO. 4-10
8	15°	NO. 0-7; NO. 1-8; NO. 2-9; NO. 3-10
9	15°	NO. 0-8; NO. 1-9; NO. 2-10
10	15°	NO. 0-9; NO. 1-10
11	15°	NO. 0-10

#### 2.5.2 Greedy strategy

The greedy algorithm operates on the principle of making the locally optimal choice at each step with the aim of achieving a globally optimal solution through a sequence of such decisions (García, 2025; Hong et al., 2025). The general procedure involves establishing a problem model, defining the greedy selection criteria, constructing the solution set, validating feasibility, and analyzing computational complexity. Unlike the conventional equidistant strategy, the greedy strategy emphasizes local optimization. In this study, the algorithm first determined the optimal position for a single camera, then, based on this selection, identified the optimal two-camera combination. For each subsequent camera, the algorithm iteratively selected the position that, when added to the previously retained stage-wise best-performing camera set, maximized overall reconstruction performance. This stepwise optimization strategy reduced the search and reconstruction burden while allowing each additional camera to be evaluated for its contribution to reconstruction performance. Using this approach, a total of 61 feasible greedy candidate configurations were evaluated (**Table 2**). The complete pseudocode, including the tie-handling and stopping rules, is provided in **Supplementary Table S3**.

**Table 2:** Stepwise camera-configuration selection using the greedy strategy.

Number of cameras	Configuration retained from previous step	Candidate cameras added (n)	Selected configuration
1	None	#0-#10 (11)	#7
2	#7	#3-#6, #8-#10 (7)	#6, #7
3	#6, #7	#2-#5, #8-#10 (7)	#4, #6, #7
4	#4, #6, #7	#0-#3, #5, #8-#10 (8)	#3, #4, #6, #7
5	#3, #4, #6, #7	#0-#2, #5, #8-#10 (7)	#3, #4, #6, #7, #8
6	#3, #4, #6, #7, #8	#0-#2, #5, #9, #10 (6)	#2, #3, #4, #6, #7, #8
7	#2, #3, #4, #6, #7, #8	#0, #1, #5, #9, #10 (5)	#2-#8
8	#2-#8	#0, #1, #9, #10 (4)	#1-#8
9	#1-#8	#0, #9, #10 (3)	#1-#9
10	#1-#9	#0, #10 (2)	#0-#9
11	#0-#9	#10 (1)	#0-#10

For each camera number, every candidate camera listed in the third column was individually added to the configuration retained from the preceding step. The final column reports the best-performing configuration among the candidates evaluated at that step. n indicates the number of candidate configurations evaluated, and camera-ID ranges are inclusive.

#### 2.5.3 Comprehensive quantitative evaluation

For each candidate configuration containing 1–10 cameras, the point cloud reconstructed using all 11 cameras was used as the corresponding internal reference. To recover its metric scale, an auxiliary point cloud was reconstructed from the corresponding original RGB images without plant segmentation, thereby retaining the cultivation pot. The reconstructed outer diameter of the pot (*d_pot_*) was measured across its outer rim in CloudCompare, and the auxiliary cloud was uniformly scaled by 33/*d_pot_*, based on the known pot outer diameter of 33 cm. The plant region of the centimeter-scaled auxiliary cloud was then used to transfer the metric scale to the plant-only 11-camera reference cloud, after which each candidate cloud was registered to the corresponding scaled reference cloud. Both the scale-transfer and candidate-to-reference registrations were performed in CloudCompare. Initial alignment was performed by manually selecting at least four pairs of corresponding feature points with the Adjust scale option enabled, followed by refinement using the iterative closest point (ICP) algorithm (**Supplementary Figure S2**). No voxel downsampling or equal-density resampling was applied before registration or distance calculation. Let P denote the 11-camera reference point cloud containing N points and Q denote the aligned candidate point cloud containing M points. For each reference point, the nearest-neighbor distance to Q was calculated using Equation 7:

(7) $${\text{d}}_{i}=\underset{q_{j}\in Q}{min}∥p_{i}-q_{j}∥$$

The ANND from the reference point cloud to the candidate point cloud was then calculated using Equation 8:

(8) $$ANND=\frac{1}{N}\sum_{i=1}^{N}{\text{d}}_{i}$$

This reference-to-candidate direction ensures that regions present in the 11-camera reference but absent from a candidate reconstruction contribute larger distances. ANND therefore represents relative geometric agreement with the internal reference, rather than absolute geometric accuracy. Total point count was retained as a descriptive indicator of point-cloud yield that indirectly reflects reconstruction completeness, rather than as an independent metric directly measuring structural completeness. Reconstruction time was defined as the total duration of COLMAP feature extraction, feature matching, and sparse reconstruction, together with OpenMVS dense reconstruction. For each camera number, reconstruction time was measured using the same three rice plants on the workstation described in Section 2.3.3 under identical software settings and summarized as the mean value (n = 3). Each run was started from scratch, and no previously generated COLMAP databases, feature files, matching results, or reconstruction outputs were reused. ANND, total point count, and reconstruction time were linearly normalized to the range 0-1. ANND and reconstruction time were treated as cost-type metrics and reverse-normalized, whereas point count was treated as a benefit-type metric. The composite score was calculated using Equation 9:

(9) $$S=w_{\text{A}}\times A+w_{\text{B}}\times B+w_{\text{C}}\times C$$

where *S* represents the composite score, *A* the accuracy score, *B* the completeness score, and *C* the efficiency score; *w*_A_, *w*_B_, and *w*_C_ are the corresponding weights, which sum to 1. Here, the accuracy score was derived from the normalized ANND and therefore represents relative geometric agreement with the internal 11-camera reference rather than absolute geometric accuracy. The completeness and efficiency scores were derived from normalized total point count and reconstruction time, respectively. Under identical reconstruction and denoising settings, total point count was used as an indirect indicator of reconstruction completeness. Accuracy, completeness, and efficiency were assigned equal weights in the comprehensive evaluation.

For metrics where lower values indicate better performance, such as average nearest-neighbor distance and reconstruction time, the normalized values were inverted to maintain consistency across all evaluation criteria. The composite scores of reconstructed point clouds under different camera configurations were then compared, and the configuration achieving the highest overall score was selected as the optimal setup.

### 2.6 Validation of point-cloud-derived morphological traits

After the optimal imaging configuration and reconstruction workflow were identified, three-dimensional point clouds of rice plants were reconstructed using the selected protocol to validate the accuracy of the point-cloud-derived morphological traits. Four rice cultivars (V1–V4) were evaluated at the heading and maturity stages. For each cultivar, three plants were selected at each growth stage, resulting in a total of 24 plant-stage observations. Immediately before image acquisition, plant height and canopy width were measured manually. Plant height was defined as the vertical distance from the soil surface in the pot to the highest point of the plant. Canopy width was measured as the maximum horizontal span of the plant at its widest position.

Following three-dimensional reconstruction and metric-scale calibration using the procedure described in Section 2.5.3, plant height was calculated as the difference between the maximum and minimum vertical coordinates of the point cloud, as shown in Equation 10:

(10) $$PH_{i}=Z_{\text{max},\;i}-Z_{\text{min},\;i}$$

where *PH_i_* is the point-cloud-derived plant height of the ith plant, and *Z*_max,*i*_ and *Z*_min,*i*_ are the maximum and minimum coordinates along the vertical axis, respectively.

Canopy width was extracted using a principal component analysis (PCA)-based oriented bounding box constrained to the horizontal plane. The point cloud was first projected onto the horizontal *XY* plane, and the principal horizontal direction was determined by eigenvalue decomposition of the covariance matrix of the projected coordinates. A second horizontal axis perpendicular to the principal direction was then established. The point-cloud extents along these two orthogonal axes were calculated, and the larger extent was defined as canopy width, as shown in Equation 11:

(11) $$CW_{i}=\max(L_{1,\;i},\;L_{2,\;i})$$

where *CW_i_* is the point-cloud-derived canopy width of the *i*th plant, and *L*_1,*i*_ and *L*_2,*i*_ are the point-cloud extents along the two orthogonal horizontal axes.

The manually measured values were used as reference observations. The agreement between the manual measurements and point-cloud-derived estimates was evaluated separately for plant height and canopy width using the coefficient of determination (R²) and root mean square error (RMSE), as defined in Equations 12, 13, respectively:

(12) $$R^{2}=\frac{{\left[\sum_{i=1}^{n}(M_{i}-\bar{M})(P_{i}-\bar{P})\right]}^{2}}{\sum_{i=1}^{n}{\left(M_{i}-\bar{M}\right)}^{2}\sum_{i=1}^{n}{\left(P_{i}-\bar{P}\right)}^{2}}$$

(13) $$RMSE=\sqrt{\frac{1}{n}\sum_{i=1}^{n}{\left(P_{i}-M_{i}\right)}^{2}}$$

where *M_i_* and *P_i_* denote the manually measured and point-cloud-derived values of the *i*th plant, respectively; $\bar{M}$ and $\bar{P}$ are the mean manually measured and point-cloud-derived values, respectively; and *n* is the number of observations.

## 3 Results

### 3.1 Performance of CBAM-UNet segmentation model

The training loss of the proposed CBAM-UNet model decreased steadily from 0.2098 at the first epoch to approximately 0.0401 at the 80th epoch, as measured by Binary Cross-Entropy with Logits Loss (**Supplementary Figure S3**). The loss curve plateaued around the 60th epoch, indicating that the model had converged. The validation-set metrics, including *mIoU*, *mPA*, and *F1*, obtained from the 60-, 70-, and 80-epoch checkpoints are summarized in **Supplementary Table S4**. These metrics showed limited variation across the three checkpoints. Based on the convergence of the training loss and the validation-set performance, the 70-epoch checkpoint was selected for subsequent analyses.

The CBAM-UNet model achieved the highest values for all three semantic segmentation metrics among the four methods evaluated (**Table 3**). Specifically, CBAM-UNet improved *mIoU* by 32.04, 0.46, and 1.12 percentage points, and *mPA* by 21.82, 0.34, and 0.65 percentage points, and *F1* by 21.39, 0.39, and 0.64 percentage points compared with thresholding, U-Net, and U-Net++, respectively (**Table 3**). In the single-run evaluation reported in **Table 3**, CBAM-UNet achieved a plant-class IoU, precision, and recall of 90.56%, 97.12%, and 93.06%, respectively. Across three independent runs, its mean *mIoU* was 93.24 ± 0.18%, compared with 92.71 ± 0.40% for U-Net. Plant-class IoU and recall were higher by 0.75 and 0.98 percentage points, respectively, whereas plant-class precision was 0.20 percentage points lower (**Supplementary Table S5**). In the seed-42 ablation, U-Net + CBAM achieved the highest *mIoU*, *mPA*, *F1*-score, plant-class IoU, and recall, whereas CAM or SAM alone did not improve *mIoU* over U-Net (**Supplementary Table S6**). Relative to U-Net, U-Net + CBAM increased the parameter count only slightly from 13.394 to 13.438 M and FLOPs from 124.17 to 124.24 G, while peak GPU memory and inference time increased from 511.4 to 667.4 MB and from 46.37 to 68.08 ms image^−^¹, respectively (**Supplementary Table S7**). Taking Hejiang18, a compact-type rice, and Guangluai4, a spreading-type rice, as examples, **Figure 3** provides a qualitative comparison of plant-structure preservation among the different segmentation methods. Thresholding yields fragmented results with substantial loss of plant structures. U-Net captures the main plant body but fails to preserve fine leaf details, resulting in omissions along leaf edges. U-Net++ improves the continuity of leaf structures relative to U-Net; however, small leaves and tips are still partially missed. In this qualitative comparison, CBAM-UNet better preserves the continuity of both major and fine plant structures, with clearly delineated leaf boundaries and fewer visually apparent missing or fragmented regions. These visual examples are intended to illustrate structural preservation, whereas the performance comparison is quantitatively supported by the *mIoU*, *mPA*, and *F1*-scores reported in **Table 3**. Using the COLMAP + OpenMVS 3D reconstruction framework, we further evaluated the effect of different image segmentation models on the completeness of 3D point clouds. Compared with Thresholding, U-Net, and U-Net++, CBAM-UNet increased the total number of reconstructed points by approximately 63%, 1%, and 2% on average, respectively (**Supplementary Table S8**). Thus, the increase relative to thresholding was substantial, whereas the gains over U-Net and U-Net++ were modest.

**Table 3:** Performance comparison of different image segmentation approaches on the validation set.

Segmentation models	mIoU (%)	mPA (%)	F1-score (%)
Thresholding	61.13	74.09	75.18
U-Net	92.71	95.57	96.18
U-Net++	92.05	95.26	95.93
CBAM-UNet	93.17	95.91	96.57

**Figure 3:**
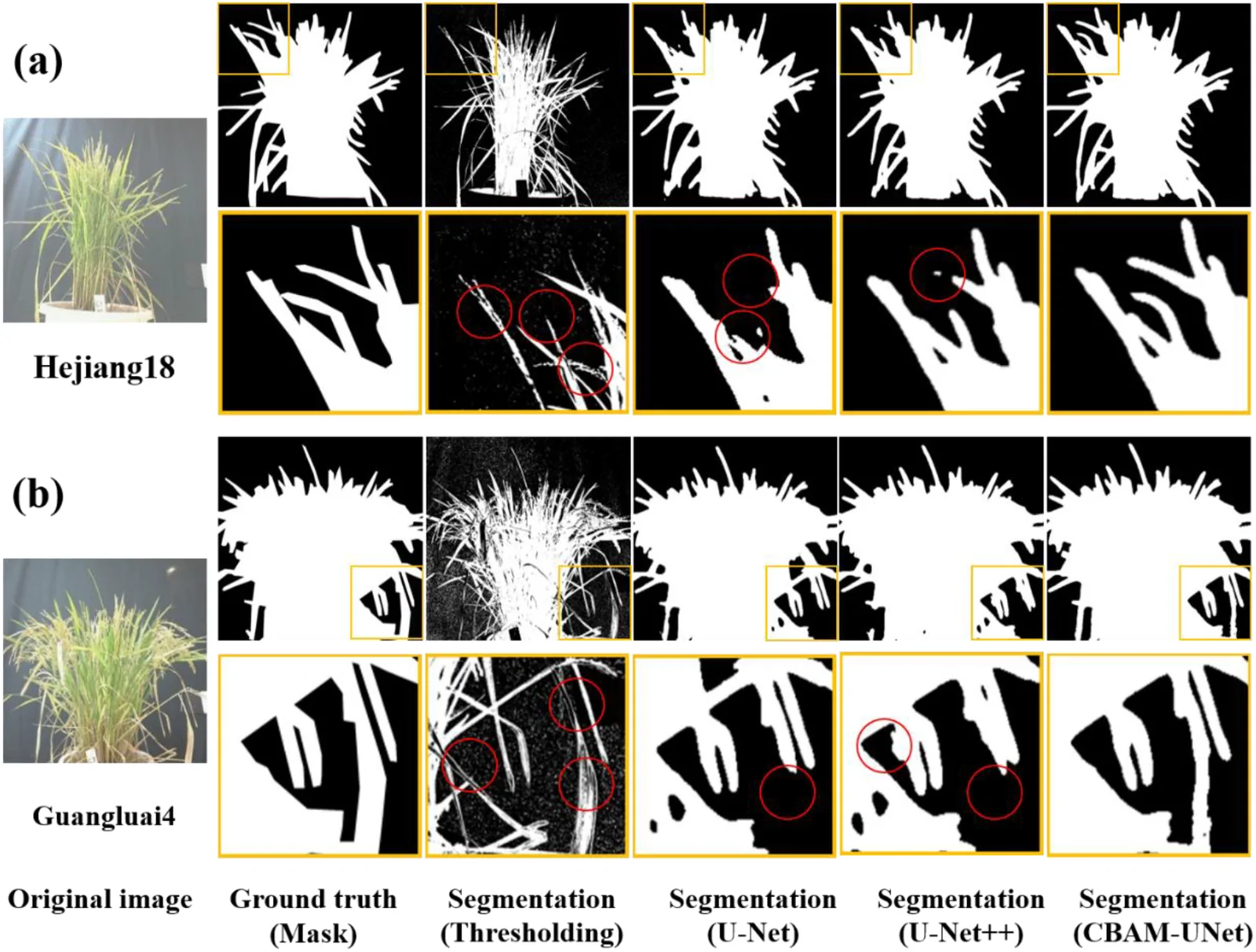
Comparison of segmentation results generated on rice plant images. **(a)** Hejiang18 (compact-type rice). **(b)** Guangluai4 (spreading-type rice). Yellow boxes highlight magnified local regions, and red circles indicate areas where segmentation errors occur. Results were obtained using four different segmentation methods: thresholding, U-Net, U-Net++, and CBAM-UNet.

### 3.2 Effect of camera configuration on 3D reconstruction

The ANNDs between the candidate point clouds and their corresponding internal reference point clouds reconstructed using all 11 cameras were analyzed. The coefficient of variation (*CV*) was high for small camera numbers, reaching 35.32% for a single camera and up to 141.35% for five-camera combinations. From six cameras onward, *CV* decreased sharply, ranging from 7.13% to 22.93% (**Figure 4a**). The median ANND decreased progressively with increasing camera number, from 0.635 cm for a single camera to 0.037 cm for ten cameras, indicating progressively closer agreement with the 11-camera internal reference (**Figure 4a**). For descriptive comparison, a reference value of 0.2 cm adopted from Hui et al. (2018) was indicated by the dashed line in **Figure 4a**. The median ANND of the single-camera configuration was above this reference value, whereas those of the other configurations were below it (**Figure 4a**).

**Figure 4:**
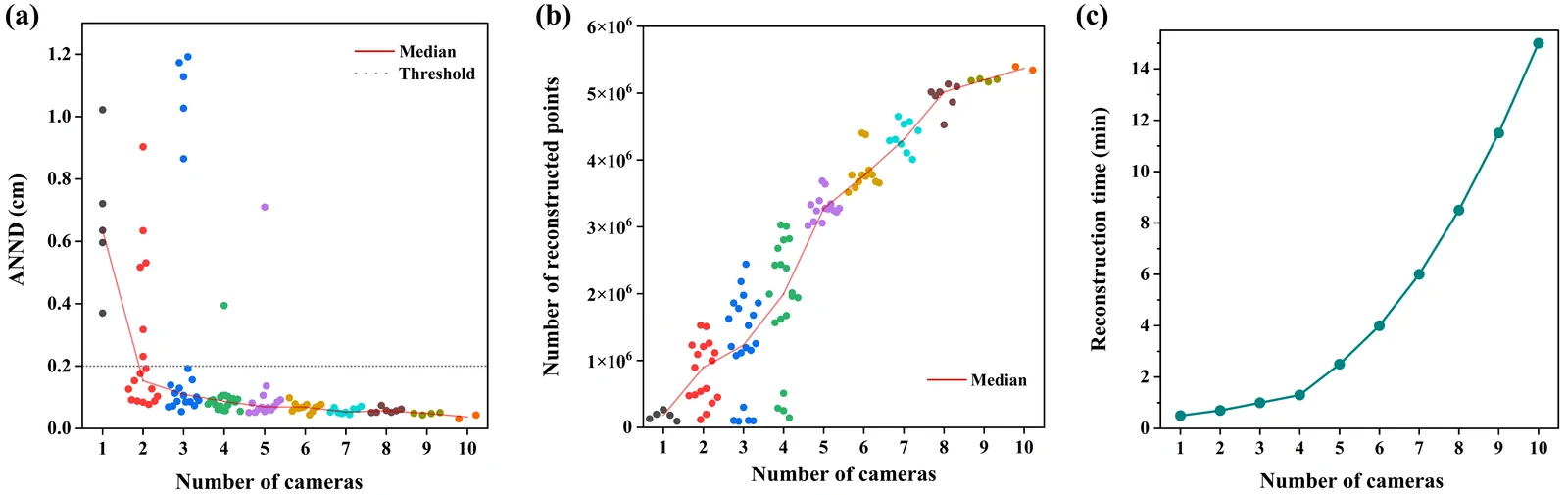
Impact of the number of cameras on 3D reconstruction accuracy, completeness, and processing time.The complete 11-camera configuration was used as the reference, and its corresponding results arereported in **Supplementary Tables S9**, **S10**. **(a)** Variation in average nearest-neighbor distance with increasing number of cameras. Different scatter points within the same camera number group represent variations in camera configuration. A threshold of 0.2 cm is indicated by the dashed line for descriptive comparison. **(b)** Variation in the number of reconstructed points with increasing number of cameras. **(c)** Variation in 3D reconstruction duration with increasing number of cameras. Each value represents the mean 3D reconstruction duration obtained from the same three rice plants under identical computational and software settings (n = 3). Timing was summarized according to the number of input cameras; therefore, configurations containing the same number of cameras share the same mean duration.

Similarly, the total point count was analyzed. The *CV* was high for small camera numbers, ranging from 0.38 for a single camera to 0.60 for three-camera combinations. From five cameras onward, *CV* decreased sharply, ranging from 0.049 to 0.007 (**Figure 4b**). The median number of points increased progressively with the number of cameras, from 182,897 for a single camera to 5,370,777 for ten cameras (**Figure 4b**). Furthermore, the mean reconstruction duration increased progressively with the number of cameras, from 0.5 min for a single camera to 15 min for ten cameras (**Figure 4c**). Because the timing results were summarized according to the number of input cameras, configurations containing the same number of cameras share the same mean reconstruction duration in **Figure 4c**.

### 3.3 Optimal camera configuration

Under both the equidistant and greedy strategies, optimal camera configurations were first evaluated based on ANND and total point count, respectively. The optimal camera IDs were identical for 1 and 7–11 cameras (**Supplementary Tables S9**, **S10**). For 2 and 3 cameras, the equidistant strategy achieved lower ANND and higher point counts than the greedy strategy. In contrast, for 4 and 5 cameras, the greedy strategy outperformed the equidistant strategy in both metrics. For 6 cameras, the equidistant strategy produced lower ANND, whereas the point count was higher under the greedy strategy (**Supplementary Tables S9**, **S10**). Subsequently, all configurations from the two strategies were combined, and the comprehensive score was used to quantify overall performance (**Table 4**). The reconstruction performance increased with the number of cameras from 1 to 6, reaching a maximum score of 0.813 at six cameras. The optimal camera configuration was #2, #3, #4, #6, #7, and #8 (**Table 4**).

**Table 4:** Optimal camera configuration determined by combining the equidistant and greedy strategies.

Number of cameras	Optimal camera configuration	Normalized average nearest-neighbor distance score	Normalized point-count score	Normalized reconstruction time score	Comprehensive score
1	NO. 7	0.000	0.000	1.000	0.333
2	NO. 4 & 5	0.792	0.238	0.989	0.673
3	NO. 3 & 4 & 5	0.854	0.410	0.973	0.746
4	NO. 3 & 4 & 6 & 7	0.851	0.521	0.957	0.776
5	NO. 3 & 4 & 6 & 7 & 8	0.862	0.645	0.892	0.800
6	NO. 2 & 3 & 4 & 6 & 7 & 8	0.849	0.780	0.811	0.813
7	NO. 2-8	0.878	0.827	0.703	0.803
8	NO. 1-8	0.876	0.919	0.568	0.788
9	NO. 1-9	0.883	0.933	0.405	0.740
10	NO. 0-9	0.916	0.968	0.216	0.700
11	NO. 0-10	1.000	1.000	0.000	0.667

Raw average nearest-neighbor distance and reconstruction time were treated as cost-type metrics, for which smaller values indicate better performance; their min–max normalized values were therefore reversed. Raw point count was treated as a benefit-type metric, for which larger values indicate better performance. Consequently, for all three normalized metric scores and the comprehensive score, a larger value indicates better performance. All normalized metric scores and comprehensive scores range from 0 to 1 and are reported to three decimal places; comprehensive scores were calculated using unrounded normalized values and rounded only for presentation.

To further validate the robustness of the optimal configuration, it was tested across four rice varieties at both the heading and maturity stages using independent data. Visualization of the ANND distribution showed that larger distances mainly occurred at the bottom of the plant and at leaf tips within the inner tillers (**Figure 5**). The results of 3D reconstruction with the optimal six-camera configuration within the evaluated candidate set showed that the ANND ranged from 0.037 to 0.066 cm relative to the corresponding 11-camera internal references, indicating close agreement across cultivars and growth stages (**Supplementary Table S11**). The total number of points reconstructed with six cameras ranged from approximately 4.073 million to 4.882 million, representing about 73.3–76.0% of the points generated using 11 cameras. Reconstruction duration with six cameras ranged from 4.0 to 5.5 min, compared to 18.0–22.0 min for 11 cameras, corresponding to a reduction of approximately 70–80% (**Supplementary Table S11**).

**Figure 5:**
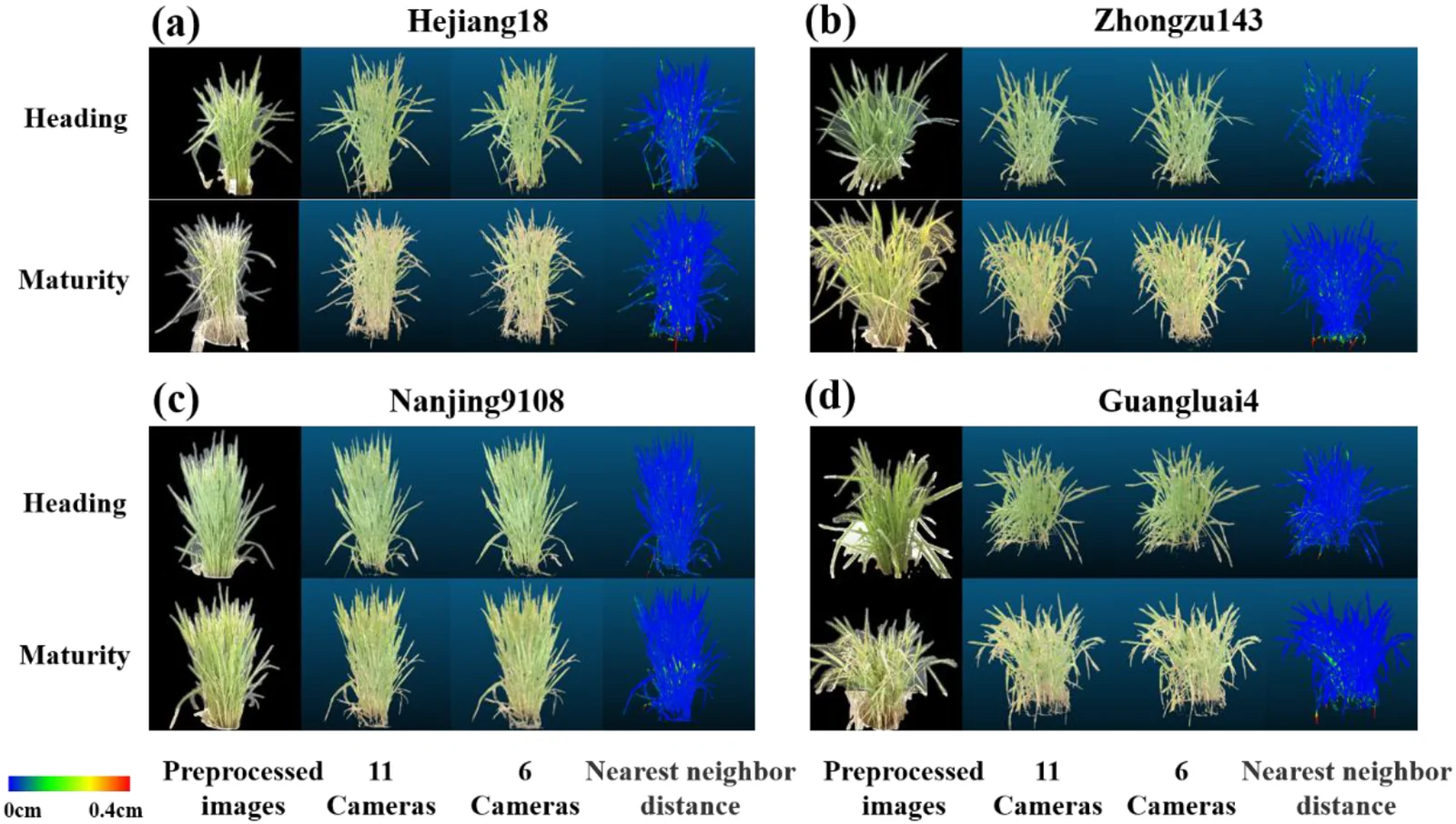
Comparison between the point cloud reconstructed using the optimal camera configuration and the reference point cloud. The reference point clouds were generated from multi-view images captured by all 11 cameras, and the average nearest-neighbor distance was calculated between each point cloud reconstructed using the optimal camera configuration and its corresponding reference. Results are shown for **(a)** Hejiang18, **(b)** Zhongzu143, **(c)** Nanjing9108, and **(d)** Guangluai4 at the heading and maturity stages. Deep-blue regions indicate smaller nearest-neighbor distances and therefore closer local agreement with the reference point cloud. A common color scale was applied across all panels to ensure direct comparability among varieties and growth stages.

### 3.4 Accuracy of phenotypic trait extraction using the optimal workflow

To evaluate the phenotypic measurement accuracy of the optimal imaging and reconstruction workflow, plant height and canopy width derived from the reconstructed point clouds were compared with the corresponding manual measurements (**Figure 6**). Point-cloud-derived plant height showed close agreement with the manually measured values, with a coefficient of determination (*R*^2^) of 0.989 and a root mean square error (*RMSE*) of 2.082 cm. The observations were distributed closely around the 1:1 line across the evaluated range of plant heights.

**Figure 6:**
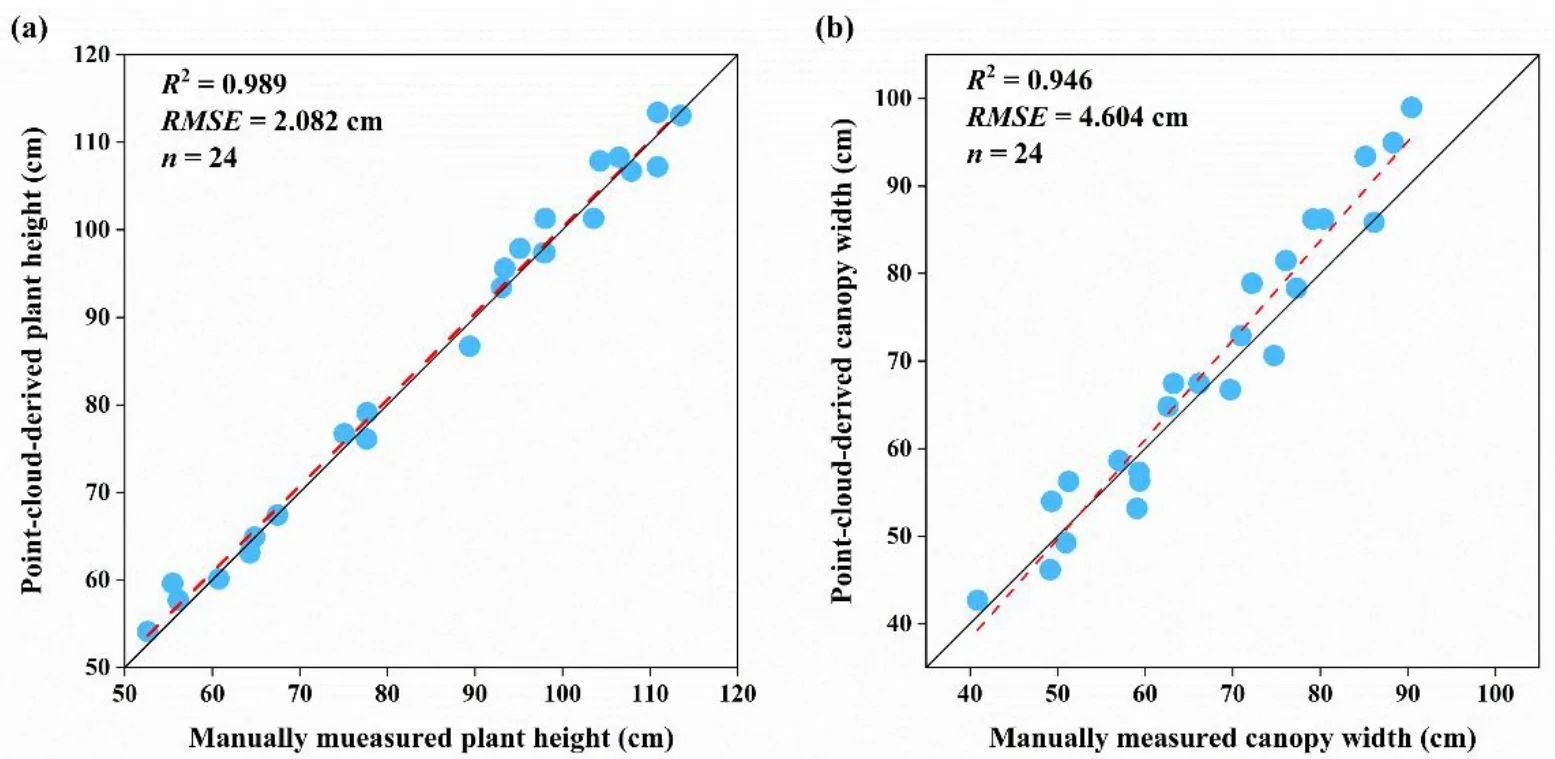
Validation of point-cloud-derived plant height and canopy width using manual measurements. Relationships between manually measured and point-cloud-derived plant height **(a)** and canopy width **(b)** obtained using the optimal imaging and reconstruction workflow. The black solid lines represent the 1:1 relationships, whereas the red dashed lines represent the fitted linear regressions.

Point-cloud-derived canopy width also showed strong agreement with the manual measurements, yielding an *R*^2^ of 0.946 and an *RMSE* of 4.604 cm. However, the canopy-width estimates exhibited greater dispersion around the 1:1 line than the plant-height estimates. Overall, the optimal workflow enabled accurate extraction of both morphological traits, with higher measurement accuracy obtained for plant height than for canopy width.

## 4 Discussion

### 4.1 Comparison of different segmentation models

Compared with thresholding, all deep learning-based segmentation methods, including U-Net, U-Net++, and CBAM-UNet, show clear advantages (**Table 3**, **Figure 3**). Thresholding relies solely on pixel intensity differences, which makes it highly sensitive to complex backgrounds, varying illumination, and subtle color variations in rice canopy images (Luo et al., 2024). In the present study, lighting conditions differed across viewpoints (**Figure 1b**), environmental backgrounds contained complex non-plant elements (**Figure 1b**), and rice color characteristics varied at different growth stages (**Figure 5**). As a result, thresholding produced fragmented segmentations and failed to maintain continuous leaf structures, leading to considerable loss of structural information. In contrast, convolutional neural networks (CNNs) such as U-Net and its variants can learn hierarchical and contextual features from training data, enabling robust segmentation under variable lighting and complex morphology, which markedly improves overall segmentation completeness (Tugrul et al., 2022).

Baseline U-Net, widely used in image segmentation, employs an encoder-decoder architecture with skip connections but has limitations in rice plant segmentation (**Figure 3**). Conventional convolution operations in U-Net do not sufficiently capture inter-channel dependencies, making it difficult to emphasize informative regions and suppress background noise, which often leads to indistinct boundaries and omission of fine leaf structures. U-Net++ introduces nested skip connections to improve structural continuity and reduce information loss, enhancing segmentation of larger structures; however, small leaves and leaf tips are still partially missed. In contrast, CBAM-UNet incorporates the CBAM into each skip connection, consisting of a CAM and a SAM. The CAM adaptively weights feature channels based on global average and max pooling, strengthening informative channels, while the SAM generates spatial attention maps by combining pooled spatial features through a convolution, guiding the network to focus on critical regions (Chen et al., 2020). This attention-guided feature fusion may contribute to the visually improved preservation of both major structures and fine details, such as leaf edges and tips, shown in **Figure 3**. The repeated-run and ablation results further showed that the combined CBAM variant achieved modest gains over U-Net in most evaluated metrics, whereas CAM or SAM alone did not improve *mIoU* (**Supplementary Tables S5**, **S6**). Together with the small gains in whole-plant point count over U-Net and U-Net++, these findings indicate an incremental rather than substantial advantage.

### 4.2 Comparison of different camera configurations

When the number of cameras was small, the variation in ANND and point count was substantial (**Figure 4**), indicating that different camera combinations strongly affected reconstruction results. For the configuration using one camera (24 azimuthal images), camera #7 at a 45° angle achieved the lowest ANND and highest point count (**Supplementary Tables S9**, **S10**), which may be related to the balance between geometric information capture and perspective provided by this viewing angle. It may allow simultaneous observation of top and side surfaces, potentially providing richer 3D cues and moderate perspective distortion that may aid depth computation. This angle may also preserve stable texture features, potentially enhancing feature matching, and reduce occlusion by covering both frontal and partial side/top surfaces. These possible explanations require further targeted validation. Similarly, in UAV-based RGB imaging with a cross-circling oblique flight route, the tilt angle is generally set to 45° to ensure comprehensive canopy coverage and robust 3D reconstruction (Xiao et al., 2023; Fei et al., 2025).

When the number of cameras increased to two, the ANND decreased sharply, falling below the 0.2 cm threshold and indicating closer agreement with the 11-camera internal reference. This trend is consistent with the benefit of complementary views reported for binocular stereo vision-based plant reconstruction (Peng et al., 2022). Across all configurations within two-camera group, camera pairs with a small inter-angle of 15° yielded the lowest ANND and the highest point count, which may be related to their greater image overlap and its potential contribution to stable feature correspondence and more reliable depth recovery (Wu et al., 2022). As the number of cameras increased, the variation among different camera combinations gradually decreased, indicating that the influence of camera arrangement became less significant than the overall number of cameras. Meanwhile, geometric agreement with the 11-camera internal reference improved and point-cloud yield increased, but at the cost of longer reconstruction duration. Therefore, applying a weighting-based evaluation approach can effectively balance accuracy, completeness, and efficiency.

The optimal camera configuration identified in this study originated from the greedy strategy. Compared with the equidistant strategy, the greedy strategy effectively avoids redundant geometric information and unstable feature matching by adaptively selecting camera angles that contribute most to reconstruction quality. In the equidistant strategy, adjacent cameras often capture highly similar perspectives, leading to redundant depth cues and limited improvements in 3D constraints. Moreover, it neglects variations in canopy structure and illumination, potentially causing feature degradation in certain views. In contrast, the greedy strategy progressively optimizes camera selection based on the reconstruction performance of preceding steps, thereby enhancing geometric complementarity while avoiding ineffective or redundant views. This adaptive optimization may have contributed to the observed favorable balance among reconstruction accuracy, completeness, and computational efficiency.

### 4.3 Robustness of the optimal imaging and reconstruction workflow

The validation across four rice cultivars and two growth stages demonstrated that the optimal imaging and reconstruction workflow was robust for quantitative phenotyping. Point-cloud-derived plant height and canopy width agreed closely with manual measurements, with *R*^2^ values of 0.989 and 0.946 and *RMSE* values of 2.082 and 4.604 cm, respectively. These results indicate that the workflow preserved the major vertical and horizontal dimensions of rice plants with different canopy architectures and developmental states.

This morphological trait validation complements the geometric and computational metrics used for camera-configuration optimization. Although nearest-neighbor distance, point count, and reconstruction time describe reconstruction quality and efficiency, they do not directly confirm the accuracy of phenotypic measurements. The lower accuracy obtained for canopy width than for plant height may be partly attributed to residual background points that were not completely removed during point-cloud denoising. Because canopy width was determined from the maximum horizontal extent of the point cloud, even a small number of residual noise points near the canopy boundary could artificially enlarge the estimated width. Overall, the selected workflow achieved a favorable balance among reconstruction quality, processing efficiency, and phenotypic measurement accuracy. Nevertheless, the validation was based on 24 potted-plant samples acquired under controlled conditions. Further evaluation using larger populations, more diverse plant architectures, and field-grown rice is needed to assess its broader applicability.

### 4.4 Remaining challenges

The use of image segmentation to remove non-plant regions prevents independent calibration with a checkerboard for multi-view imaging. We rely on periodic calibration to determine the intrinsic and extrinsic parameters of the cameras. However, slight shifts in camera positions or angles may occur during rotation, and periodic checkerboard calibration may not fully capture these deviations, potentially introducing errors in the world coordinate system and affecting subsequent phenotypic measurements. To improve calibration reliability and enable evaluation of absolute geometric accuracy, it would be necessary to retain checkerboard or other reference markers alongside the plant pixels during segmentation, enabling accurate calibration without compromising plant feature extraction.

For rice individuals with dense tillers and severe leaf overlap, internal regions of the canopy, particularly the lower leaves, still exhibit holes or noisy points in the reconstructed point clouds (**Figure 5**), indicating incomplete or noisy reconstruction in these areas. This reduction in local point-cloud quality is mainly due to occlusion and the restricted camera angles available to capture the bottom of the canopy. Point cloud completion techniques can partially address these gaps (Chen et al., 2023; Li et al., 2025), but their effectiveness depends on assumptions about plant structure and may introduce artifacts, so improving internal point cloud quality remains a key challenge.

The current camera configuration optimization was conducted using our self-developed 3D phenotyping platform, Metatlas V1. Since this optimization was specifically designed for the geometry, sensor properties, and imaging workflow of this system, it is uncertain whether the resulting optimal configuration can be directly applied to other multi-view imaging platforms, including UAV-based systems, handheld multi-camera arrays, or fixed field-based camera arrays. In addition, the study focused solely on rice. Crops with substantially different morphologies, such as tall maize with relatively simple canopy structures or shorter soybean with dense canopies, may produce different reconstruction outcomes. Consequently, the generalizability of the optimized camera configuration to other platforms and plant species remains uncertain and warrants further systematic evaluation. Nevertheless, the evaluation framework used here may provide a methodological reference for other multi-view systems by jointly considering relative geometric agreement, point-cloud yield, and computational efficiency.

## 5 Conclusions

In this study, we developed an optimized workflow for high-throughput 3D reconstruction of rice plants using the Metatlas V1 multi-view imaging platform. By integrating CBAM-UNet-based image segmentation with a comprehensive camera configuration optimization, which fused equidistant and greedy strategies, we achieved precise and efficient reconstruction of rice plants.

Compared with conventional thresholding, CBAM-UNet improved *mIoU* by 32.04 percentage points, mPA by 21.82 percentage points, and F1 by 21.39 percentage points. Its improvements over U-Net and U-Net++ were more modest, ranging from 0.46–1.12, 0.34–0.65, and 0.39–0.64 percentage points for *mIoU*, *mPA*, and *F1*, respectively. The model accurately delineated both major and fine leaf structures, preserving continuous leaf edges and tips while minimizing fragmented or missing regions. When used for 3D reconstruction based on segmented multi-view images, CBAM-UNet also yielded higher point counts compared with other models.

Camera configuration had a substantial impact on reconstruction performance. Increasing the number of cameras reduced the ANND and increased point count, while reconstruction time also increased. By combining equidistant and greedy strategies, an optimal six-camera configuration (#2, #3, #4, #6, #7, and #8, corresponding to −30°, −15°, 0°, +30°, +45°, and +60° relative to the horizontal viewpoint) was identified. This setup achieved a low ANND of 0.037–0.066 cm, retained 73–76% of the points produced by the full 11-camera arrangement, and reduced reconstruction duration by approximately 70–80%, effectively balancing reconstruction accuracy, point cloud completeness, and computational efficiency.

## Data Availability

Eight representative 3D point clouds covering four rice cultivars at the heading and maturity stages, together with the phenotypic trait extraction code and CBAM-UNet source code, are publicly available on Zenodo at https://doi.org/10.5281/zenodo.22016319. Additional data can be obtained from the corresponding author upon reasonable request.
