# Supplementary material for: Balancing accuracy, completeness, and efficiency for rice 3D reconstruction through CBAM-UNet-based multi-view segmentation and camera configuration optimization
Source: Front Plant Sci. 2026 Sep 24;17:1882534. doi: 10.3389/fpls.2026.1882534 (PMC13646141; doi:10.3389/fpls.2026.1882534)
Supplement: Supplementary file 1 [file DataSheet1.docx]

Supplementary Materials

**Contents**

Tables S1 to S11

Figs. S1 to S3

Table S1. Correspondence between camera position numbers and viewing angles in the 11-camera configuration of the Metatlas V1 platform

| **Camera position index** | **Viewing angle (°)** |
| --- | --- |
| 0 | -60 |
| 1 | -45 |
| 2 | -30 |
| 3 | -15 |
| 4 | 0 |
| 5 | 15 |
| 6 | 30 |
| 7 | 45 |
| 8 | 60 |
| 9 | 75 |
| 10 | 90 |

***Note:*** Camera positions were indexed from 0 to 10 (11 positions in total). Viewing angles were measured relative to the horizontal direction (0°), with negative and positive values indicating downward- and upward-facing orientations, respectively.

Table S2. Composition of the image segmentation dataset by cultivar, growth stage, plant identity, horizontal viewing position, and camera position

| **Cultivar** | **Growth stage** | **Training plants** | **Training images** | **Validation plant** | **Validation images** |
| --- | --- | --- | --- | --- | --- |
| V1 | Heading | R1–R5 | 60 | R6 | 15 |
| V1 | Maturity | R1–R5 | 60 | R6 | 15 |
| V2 | Heading | R1–R5 | 60 | R6 | 15 |
| V2 | Maturity | R1–R5 | 60 | R6 | 15 |
| V3 | Heading | R1–R5 | 60 | R6 | 15 |
| V3 | Maturity | R1–R5 | 60 | R6 | 15 |
| V4 | Heading | R1–R5 | 60 | R6 | 15 |
| V4 | Maturity | R1–R5 | 60 | R6 | 15 |

***Note:*** V1–V4 denote Hejiang18, Zhongzu143, Nanjing9108, and Guangluai4, respectively, and R1–R6 denote individual plants within each cultivar. For each cultivar–stage combination, the 60 training images comprised 55 images of R1–R5 acquired at one randomly selected horizontal viewing position using all 11 vertical camera positions (#0–#10), together with five additional images of R1 acquired at a second horizontal viewing position using camera positions #1, #3, #4, #6, and #9. The 15 validation images comprised 11 images of R6 acquired at one horizontal viewing position using positions #0–#10 and four additional images acquired at a second horizontal viewing position using positions #1, #4, #6, and #9.

Table S3. Pseudocode for the stepwise greedy search of camera configurations

| **Input:** *C* = {#0, #1, ..., #10}; elevation angle *θ*(*c*); maximum angular gap *Δ*_max_ = 60°; and the 11-camera reference point cloud *P*_ref_. | |
| --- | --- |
| **Output:** *Ω_G_*, the set of all configurations evaluated during the greedy search; and *S_k_*, the stage-wise best-performing configuration retained for each camera number *k*. | |
| 1 | Set *S*_0_ ← ∅ and *Ω_G_* ← ∅. |
| 2 | **for** *k* = 1 to 11 **do** |
| 3 | Initialize the feasible candidate set *A_k_* ← ∅. |
| 4 | **for each** camera *c* ∈ *C* \ *S_k_*_−1_ **do** |
| 5 | Set *S′* ← *S_k_*_−1_ ∪ {*c*} and sort the cameras in *S′* by elevation angle. |
| 6 | **if** *k* = 1, or every angular gap between adjacent selected cameras in *S′* is ≤ *Δ*_max_, **then** |
| 7 | Add *S′* to *A_k_*. |
| 8 | **end if** |
| 9 | **end for** |
| 10 | **for each** candidate configuration *S′* ∈ *A_k_* **do** |
| 11 | Reconstruct its point cloud *P*(*S′*). |
| 12 | Calculate *d*(*S′*), the average nearest-neighbor distance between *P*(*S′*) and *P*_ref_. |
| 13 | Calculate *n*(*S′*), the total number of points in *P*(*S′*). |
| 14 | **end for** |
| 15 | Calculate *A*(*S′*) = [*d*_max,_*_k_* − *d*(*S′*)] / [*d*_max,_*_k_* − *d*_min,_*_k_*], the reversed min-max normalized distance score within *A_k_*. |
| 16 | Calculate *B*(*S′*) = [*n*(*S′*) − *n*_min,_*_k_*] / [*n*_max,_*_k_* − *n*_min,_*_k_*], the min-max normalized point-count score within *A_k_*. |
| 17 | Calculate the stage-wise score *G*(*S′*) = 0.5*A*(*S′*) + 0.5*B*(*S′*). |
| 18 | Select *S_k_* ← arg max {*G*(*S′*) : *S′* ∈ *A_k_*}. |
| 19 | **If** scores are tied, retain the candidate with the lower *d*(*S′*); **if** the tie remains, retain the candidate with the larger *n*(*S′*); **if** it still remains, retain the lexicographically smaller camera-ID combination. |
| 20 | Update *Ω_G_* ← *Ω_G_* ∪ *A_k_*. |
| 21 | **if** *S_k_* = *C* **then** |
| 22 | Stop the search. |
| 23 | **end if** |
| 24 | **end for** |
| 25 | **return** *Ω_G_* and {*S*_1_, *S*_2_, ..., *S*_11_}. |

***Note.*** Because reconstruction time was identical among configurations containing the same number of cameras, it did not discriminate among candidates within an individual greedy-selection step. Therefore, the stage-wise objective used equally weighted normalized mean nearest-neighbor distance and point count, whereas reconstruction time was included in the subsequent comprehensive evaluation across different camera numbers. The numbers of feasible candidate configurations evaluated for *k* = 1–11 were 11, 7, 7, 8, 7, 6, 5, 4, 3, 2, and 1, respectively, yielding 61 tested greedy candidates in total.

Table S4. CBAM-UNet validation-set metrics at different epochs.

| **Number of epochs** | **mIoU (%)** | | **mPA (%)** | **F1-score (%)** |
| --- | --- | --- | --- | --- |
| 60 | | 92.96 | 95.67 | 96.36 |
| 70 | | 93.17 | 95.91 | 96.57 |
| 80 | | 92.61 | 95.75 | 96.15 |

Table S5. Performance of U-Net and CBAM-UNet across three independent training runs

| **Model** | **mIoU** **(%)** | **mPA** **(%)** | **F1-score** **(%)** | **Plant IoU** **(%)** | **Plant precision** **(%)** | **Plant recall** **(%)** |
| --- | --- | --- | --- | --- | --- | --- |
| U-Net | 92.71 ± 0.40 | 95.62 ± 0.30 | 96.19 ± 0.22 | 89.92 ± 0.56 | 96.95 ± 0.31 | 92.54 ± 0.67 |
| CBAM-UNet | 93.24 ± 0.18 | 96.05 ± 0.18 | 96.48 ± 0.10 | 90.67 ± 0.26 | 96.75 ± 0.32 | 93.52 ± 0.49 |

***Note:*** Values are presented as the mean ± SD of three independent training runs, comprising the original randomly initialized run and two additional runs with fixed random seeds (42 and 123). *mIoU*, *mPA*, and *F1*-score were macro-averaged across the background and plant classes. Plant-class metrics were calculated from the aggregated pixel-level confusion matrix.

Table S6. Single-seed ablation results of the attention modules

| **Model** | **mIoU** **(%)** | **mPA** **(%)** | **F1-score** **(%)** | **Plant IoU** **(%)** | **Plant precision** **(%)** | **Plant recall** **(%)** |
| --- | --- | --- | --- | --- | --- | --- |
| U-Net | 93.01 | 95.78 | 96.36 | 90.34 | 97.21 | 92.75 |
| U-Net + CAM | 92.87 | 95.62 | 96.28 | 90.13 | 97.40 | 92.35 |
| U-Net + SAM | 92.39 | 95.21 | 96.02 | 89.44 | 97.67 | 91.39 |
| CBAM-UNet | 93.44 | 96.26 | 96.59 | 90.96 | 96.53 | 94.04 |

***Note:*** All models were trained and evaluated using the same dataset partition, training protocol, and fixed random seed (42). *mIoU*, *mPA*, and *F*1-score were macro-averaged across the background and plant classes. CAM and SAM were inserted into the four skip connections before the decoder, while CBAM sequentially combined channel and spatial attention.

Table S7. Computational complexity and inference efficiency of the evaluated models

| **Model** | **Parameters** **(M)** | **FLOPs** **(G)** | **Peak GPU memory** **(MB)** | **Inference time** **(ms image⁻¹)** |
| --- | --- | --- | --- | --- |
| U-Net | 13.394 | 124.17 | 511.4 | 46.37 |
| U-Net++ | 24.829 | 534.99 | 1587.9 | 201.95 |
| U-Net + CAM | 13.438 | 124.21 | 616.1 | 65.36 |
| U-Net + SAM | 13.395 | 124.21 | 563.4 | 49.33 |
| U-Net + CBAM | 13.438 | 124.24 | 667.4 | 68.08 |

***Note:*** Model size was represented by the number of trainable parameters. FLOPs were calculated for one forward pass with a 512 × 512 input and a batch size of 1. Peak GPU memory was measured using torch.cuda.max_memory_allocated(). Mean inference time was calculated from 100 synchronized forward passes after 10 warm-up passes on an NVIDIA RTX 4070 GPU. Image loading and preprocessing were excluded.

Table S8. Point counts for different rice varieties reconstructed from multi-view images processed using various image segmentation models

| Variety | Thresholding | U-Net | U-Net++ | CBAM-UNet |
| --- | --- | --- | --- | --- |
| Hejiang18 | 3172135 | 5227243 | 5151728 | 5273276 |
| Zhongzu143 | 3428379 | 5586713 | 5512720 | 5628352 |
| Nanjing9108 | 3037196 | 4805278 | 4783203 | 4875863 |
| Guangluai4 | 3823065 | 6053701 | 5982618 | 6127385 |

Table S9. Optimal camera configuration under the equidistant strategy based on average nearest-neighbor distance and point count

| **Number of cameras** | **Optimal camera IDs, average nearest-neighbor distance (cm)** | **Optimal camera IDs, number of reconstructed points** | **Reconstruction duration (min)** |
| --- | --- | --- | --- |
| 1 | NO. 7, 0.37 | NO. 7, 265603 | 0.5 |
| 2 | NO. 4 & 5, 0.077 | NO. 4 & 5, 1528634 | 0.7 |
| 3 | NO. 3 & 4 & 5, 0.054 | NO. 3 & 4 & 5, 2441399 | 1 |
| 4 | NO. 2-5, 0.061 | NO. 3-6, 3008888 | 1.3 |
| 5 | NO. 3-7, 0.059 | NO. 3-7, 3637756 | 2.5 |
| 6 | NO. 2-7, 0.044 | NO. 3-8, 4378982 | 4 |
| 7 | NO. 2-8, 0.045 | NO. 2-8, 4650590 | 6 |
| 8 | NO. 1-8, 0.046 | NO. 1-8, 5135602 | 8.5 |
| 9 | NO. 1-9, 0.043 | NO. 1-9, 5210871 | 11.5 |
| 10 | NO. 0-9, 0.031 | NO. 0-9, 5397832 | 15 |
| 11 | NO. 0-10, 0 | NO. 0-10, 5567146 | 19 |

Table S10. Optimal camera configuration under the greedy strategy based on average nearest-neighbor distance and point count

| **Number of cameras** | **Optimal camera IDs, average nearest-neighbor distance (cm)** | **Optimal camera IDs, number of reconstructed points** | **Reconstruction duration (min)** |
| --- | --- | --- | --- |
| 1 | NO. 7, 0.37 | NO. 7, 265603 | 0.5 |
| 2 | NO. 6 & 7, 0.083 | NO. 6 & 7, 1231020 | 0.7 |
| 3 | NO. 4 & 6 & 7, 0.069 | NO. 4 & 6 & 7, 1936690 | 1 |
| 4 | NO. 3 & 4 & 6 & 7, 0.055 | NO. 3 & 4 & 6 & 7, 3028131 | 1.3 |
| 5 | NO. 3 & 4 & 6 & 7 & 8, 0.051 | NO. 3 & 4 & 6 & 7 & 8, 3685813 | 2.5 |
| 6 | NO. 2 & 3 & 4 & 6 & 7 & 8, 0.056 | NO. 2 & 3 & 4 & 6 & 7 & 8, 4402186 | 4 |
| 7 | NO. 2-8, 0.045 | NO. 2-8, 4650590 | 6 |
| 8 | NO. 1-8, 0.046 | NO. 1-8, 5135602 | 8.5 |
| 9 | NO. 1-9, 0.043 | NO. 1-9, 5210871 | 11.5 |
| 10 | NO. 0-9, 0.031 | NO. 0-9, 5397832 | 15 |
| 11 | NO. 0-10, 0 | NO. 0-10, 5567146 | 19 |

Table S11. Performance of 3D reconstruction across various plant architectures and growth stages using the optimal camera configuration

| **Variety** | **Growth stage** | **Number of cameras** | **Average nearest-neighbor distance (cm)** | **Number of reconstructed points** | **Reconstruction duration (min)** |
| --- | --- | --- | --- | --- | --- |
| Hejiang  18 | Heading | 6 | 0.055 | 4074535 | 4 |
|  |  | 11 |  | 5049330 | 20 |
|  | Maturity | 6 | 0.066 | 4236130 | 4 |
|  |  | 11 |  | 5582254 | 20 |
| Zhongzu  143 | Heading | 6 | 0.048 | 4364649 | 5 |
|  |  | 11 |  | 5536503 | 20 |
|  | Maturity | 6 | 0.062 | 4586408 | 5 |
|  |  | 11 |  | 5543535 | 20 |
| Nanjing  9108 | Heading | 6 | 0.037 | 4166156 | 4 |
|  |  | 11 |  | 5016539 | 18 |
|  | Maturity | 6 | 0.049 | 4203685 | 4 |
|  |  | 11 |  | 5030473 | 18 |
| Guangluai  4 | Heading | 6 | 0.052 | 4882520 | 5.5 |
|  |  | 11 |  | 6461517 | 22 |
|  | Maturity | 6 | 0.065 | 4767410 | 5.5 |
|  |  | 11 |  | 6624560 | 22 |

***Note:*** *Values represent the means of three biological replicates for each cultivar–stage combination (n = 3).*

Figure S1. Manual annotation and label configuration of the multi-view rice image dataset. **a.** Polygonal masks were drawn around each rice plant to precisely delineate plant regions from the background. **b.** The images were manually annotated using the LabelMe software. The annotated images were then converted into binary masks, where pixel values corresponding to rice plant regions were assigned 1, and those of the background were assigned 0.

Figure S2. Workflow for point-cloud registration and ANND calculation. **a.** The denoised 11-camera reference point cloud (left) and candidate point cloud (right) before registration. **b.** Manual selection of corresponding feature-point pairs for initial scale-adjusted alignment. **c.** Refined registration using the iterative closest point (ICP) algorithm, with the two aligned point clouds displayed in contrasting colors. **d. Visualization of the reference-to-candidate nearest-neighbor distances after registration; the color scale represents distances ranging from 0 to 0.4 cm.**

Figure S3. Training loss curve of the CBAM-UNet model. Pixel-wise classification errors between predicted and ground-truth masks were computed using Binary Cross-Entropy with Logits Loss. The dashed line indicates the loss value at the 60th epoch, where the curve stabilized.
